# A Prospective Study and Single-Center Experience: Effectivity of Fusion Prostate Biopsy in Biopsy-Naïve Patients

**DOI:** 10.7759/cureus.19002

**Published:** 2021-10-24

**Authors:** Türev Demirtaş, Ahmet Gur, Abdullah Golbasi, Gökhan Sönmez, Şevket T Tombul, Abdullah Demirtaş

**Affiliations:** 1 History of Medicine and Ethics, Erciyes University, Kayseri, TUR; 2 Urology, Kayseri City Hospital, Kayseri, TUR; 3 Urology, Erciyes University, Kayseri, TUR

**Keywords:** combined biopsy, targeted biopsy, prostate cancer, magnetic resonance imaging, clinically significant cancer

## Abstract

Objective

Fusion prostate biopsy (FPB) has become a popular technique in biopsy-naïve patients, though not accepted as a standard approach (yet). In this study, we aimed to present the clinical outcomes of biopsy-naïve patients who underwent FPB.

Material and methods

The study included 400 biopsy-naïve patients aged 45-75 years who had a prostate-specific antigen (PSA) level of 2-10 ng/ml and were detected with a Prostate Imaging-Reporting and Data System (PIRADS) ≥3 lesion on multiparametric prostate magnetic resonance imaging (mpMRI)-guided FPB. A combined biopsy (CB) was performed in each patient, in which 2-4 cores were obtained for suspicious lesions by targeted biopsy (TB) and then 12-core standard prostate biopsy (SPB) was conducted in the same session. Cancer detection rates, clinically significant prostate cancer (csPCa) detection rates, histological upgrading rates, and false negative rates were determined.

Results

The 400 patients had a mean age of 62.01±7.00 years and a mean PSA value of 6.84±1.87 ng/ml. Overall PCa detection rate was 50% (200/400). The csPCa detection rates for TB, SPB, and CB were 25.0%, 31.8%, and 44.0%, respectively (p<0.001). In PIRADS 3, 4, and 5 lesions, CB had a csPCa detection rate of 29.2%, 54%, and 64.8%, respectively (p<0.001). The ratio of false negativity was significantly higher for TB compared to SPB (43.2% vs. 27.8%, p=0.003), whereas no significant difference was found between these two techniques with regard to upgrading rates although TB had a higher rate (19.6% vs. 13.7%, p=0.144).

Conclusion

FPB, a combined approach involving TB and SPB, was revealed as the most successful technique in biopsy-naïve patients with PSA<10 ng/ml due to its high cancer detection rates and low false negative rates.

## Introduction

Prostate cancer (PCa) is the leading urological cancer worldwide [1]. Fusion prostate biopsy (FPB) has recently emerged as a popular and important tool in the diagnosis of PCa [2]. FPB can be applied in the form of targeted biopsy (TB) which is based only on obtaining specimens from suspicious lesions or in combined biopsy (CB) in which 10- to 12-core standard prostate biopsy (SPB) is applied in addition to TB [3].

In the early 2010s, multiparametric prostate magnetic resonance imaging (mpMRI)-guided FPB was recommended only for patients with a history of negative biopsy and for patients with ongoing cancer suspicion [4-6]. In recent years, however, FPB has been shown to be an effective method also in biopsy-naïve patients who have moderate prostate-specific antigen (PSA) levels (<10 ng/ml) but have ongoing cancer suspicion. Thanks to FPB, although it varies according to the character of the lesion, up to 60% of cancer can be diagnosed [7-9].

In one of our previous studies, we prospectively evaluated our early results of 80 patients and we found that the FPB administered in CB could be a successful technique in biopsy-naïve patients [10]. In the present study, we aimed to evaluate clinical outcomes of another cohort of 400 biopsy-naïve patients with a PSA level of 2-10 ng/ml.

## Materials and methods

Patient selection and data collection

The prospective study included patients that underwent FPB (TB + SPB) due to elevated age-adjusted PSA levels and/or suspicious digital rectal examination (DRE) findings. Inclusion criteria were PSA 2-10 ng/ml, absence of a history of biopsy, a Prostate Imaging-Reporting and Data System (PIRADS) 3 or higher lesion detected on mpMRI, and age between 40-75 years. Patients with a history of biopsy and PIRADS <3 lesions were excluded from the study.

Age, PSA value, DRE findings, body mass index (BMI) which calculated according to the formula kg/m^2^, family history of PCa, and comorbidities were recorded for each patient. SPB, TB, and CB were compared with regard to cancer detection rates and cancer stages and TB and SPB were compared in terms of false negative and upgrading rates. The definition of "upgrade" was accepted as an increase in the grade of Gleason or the transformation of the cancer from unilateral to bilateral.

Multiparametric prostate magnetic resonance imaging (mpMRI)

Prior to biopsy procedure, an mpMRI scan was performed in each patient using a 1.5 T MRI device (Siemens Magnetom-1.5 Tesla-Siemens Medical Solutions, PA, USA). Suspicious lesions detected on diffusion-weighted images (DWI) and contrast-enhanced T2-, T1-weighted images were recorded according to PIRADS version 2 [11]. In patients detected with multiple lesions with different PIRADS scores, the lesion having the highest PIRADS score was accepted as the dominant lesion.

Fusion prostate biopsy

A sterile urine culture was obtained from each patient prior to the biopsy procedure. As long as there was no obstacle, routine prebiopsy ciprofloxacin prophylaxis was applied to the patients. All the FPB procedures were performed with local anesthesia or sedoanalgesia under outpatient clinic conditions and completed by the same urologists (AD, STT, GS). Transrectal biopsies were performed using an ultrasonography (USG) system with rigid fusion software (LOGIQ-E9, General Electric, MA, USA).

Subsequently, the mpMRI images were transferred to the USG system and then were synchronized with USG images. Total prostate volume was assessed based on the height (H), width (W), and length (L) of the prostate, using the following formula: H × W × L × 0.523 (mm^3^). TB was performed by obtaining 2-4 cores from suspicious lesions detected at the beginning. After completing the TB procedure, synchronization was discontinued (i.e. blind mpMRI) and 12 additional cores were obtained from the same patient, thereby performing FPB in the form of CB.

Each specimen was sent for histopathological examination in separate tubes. Patients reported with high-grade prostatic intraepithelial neoplasm (HGPIN) in three or more cores or atypical small acinar proliferation (ASAP) were referred to rebiopsy and were included to the benign group during the analysis. The percentage of positive cores in biopsy was recorded according to primary and secondary Gleason score and the International Society of Urological Pathology (ISUP) grading system for PCa [12]. Clinically significant prostate cancer (csPCa) was considered as Gleason score ≥3+4 or maximum cancer core length ≥5 mm [13].

Statistical analysis

Data were analyzed using SPSS 22.0 for Windows (IBM Corp., Armonk, USA). Normal distribution of data was assessed using Shapiro-Wilk test and histogram plots. Variables with normal distribution were expressed as mean ± standard deviation (SD). Among variables with nonnormal distribution, continuous variables were expressed as median (1st-3rd quartile) and categorical variables were expressed as percentages (%). Among multiple dependent groups, continuous variables with normal distribution were compared using Repeated Measures ANOVA, continuous variables with nonnormal distribution were compared using Friedman’s test, and categorical variables were compared using Cochran’s Q test. Categorical variables were compared between two dependent groups using McNemar’s test. A p-value of <0.05 was considered significant.

Ethical approval

The study protocol was approved by the Ethics Committee of the Medical Faculty of Erciyes University (Approval No. 2014/508). An informed consent was obtained from each patient.

## Results

The 400 patients had a mean age of 62.01 ± 7.00 years and a mean PSA value of 6.84 ± 1.87 ng/ml. A PIRADS 3 lesion was detected in 48% while PIRADS 4 and 5 lesions were detected in 34.2% and 17.8% of the patients, respectively. CB had a total PCa detection rate of 50% and a csPCa detection rate of 44% (Table 1).

**Table 1:** Demographic and clinical characteristics BMI: Body mass index, PSA: Prostate-specific antigen, PIRADS: Prostate Imaging-Reporting and Data System, PCa: Prostate cancer, ISUP: International Society of Urological Pathology

Variable	Value (n = 400)
Age (years)	62.01 ± 7.00
BMI (m^2^/kg)	27.72 ± 3.44
PSA (ng/ml)	6.84 ± 1.87
Family history of prostate cancer (n, %)	96/400 (24.0%)
Size of the transition zone (mm^3^)	30.74 (18.18-45.98)
Total prostate volume (mm^3^)	52.25 (39.16-72.53)
Anesthetic technique (n, %)	
Local	292 (73.0%)
Sedoanalgesia	108 (27.0%)
PIRADS score (n, %)	
3	192 (48.0%)
4	137 (34.2%)
5	71 (17.8%)
Total number of quadrants	14.0 (13.0-14.0)
Number of targeted quadrants	3.0 (2.0-3.0)
Total number of cores	19.0 (17.0-19.0)
Targeted cores	7.0 (5.0-7.0)
Clinically significant PCa (n, %)	176 (44.0%)
Total PCa rate (n, %)	200 (50.0%)
ISUP score (n, %)	
1	92 (52.6%)
2	29 (16.6%)
3	16 (9.1%)
4	25 (14.3%)
5	14 (8.0%)

**Figure 1:**
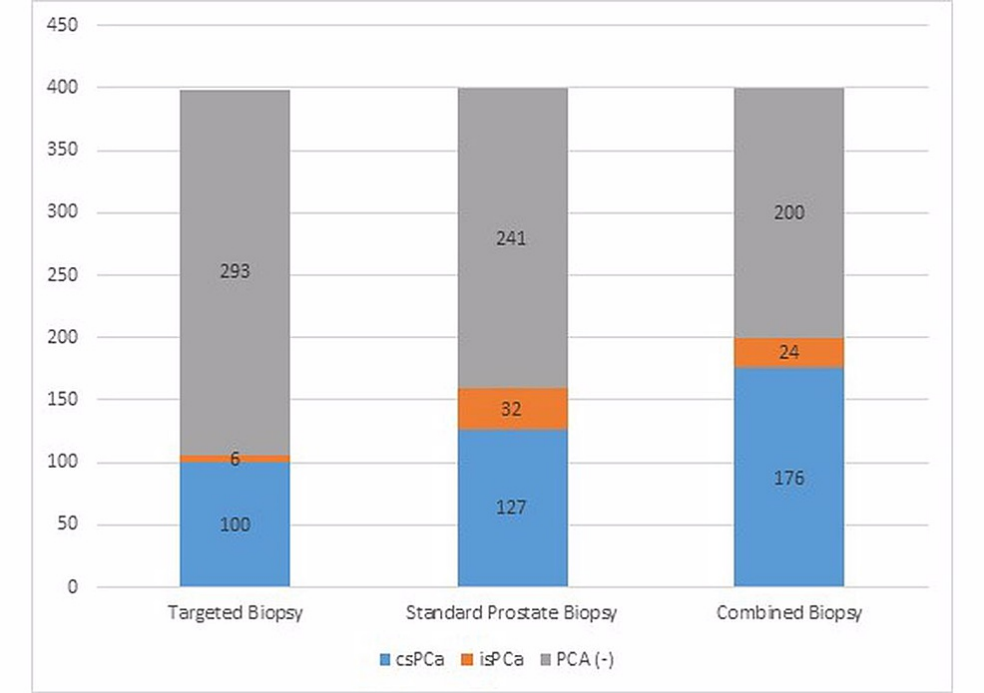
Clinically significant prostate cancer detection rates (p-values for: TB/SPB = 0.009, TB/CB = 0.061, SPB/CB = 0.032). PCa: Prostate cancer, csPCa: Clinically significant prostate cancer, isPCa: Insignificant prostate cancer, SPB: Standard prostate biopsy, TB: Targeted biopsy, CB: Combined biopsy.

Among all three techniques, SPB had the highest clinically insignificant PCa (isPCa) detection rate (Figure 1).

Cancer detection rates according to biopsy techniques and PIRADS scores

In PIRADS 3 lesions, the csPCa detection rate was 10.4% for TB, 20.8% for SPB, and 29.2% for CB (p<0.001). In PIRADS 4 lesions, the csPCa detection rate was similar for TB and SPB (30.7% and 34.3%, respectively) while it was significantly higher for CB (54%, p<0.001). In PIRADS 5 lesions, the csPCa detection rates were similar to those of PIRADS 4 lesions and the highest rate was detected for CB. Table 2 presents the csPCa rates according to PIRADS scores and biopsy techniques.

**Table 2:** Cancer detection rates in targeted biopsy, standard prostate biopsy, and combined biopsy according to PIRADS scores PIRADS: Prostate Imaging-Reporting and Data System

	Targeted biopsy	Standard Prostate Biopsy	Combined Biopsy	p
PIRADS 3 (n=192)	20 (10.4%)	40 (20.8%)	56 (29.2%)	<0.001
PIRADS 4 (n=137)	42 (30.7%)	47 (34.3%)	74 (54.0%)	<0.001
PIRADS 5 (n=71)	38 (53.5%)	40 (56.3%)	46 (64.8%)	0.024
Overall (n=400)	100 (25.0%)	127 (31.8%)	176 (44.0%)	<0.001

False negativity and upgrading rates

The ratio of false negativity was significantly higher for TB compared to SPB (43.2% vs. 27.8%, p=0.003). In patients detected with csPCa, although the overall upgrading rate was higher for TB compared to SPB, no significant difference was established (19.6% vs. 13.7%, p=0.144). Table 3 presents the ISUP scores for each biopsy technique.

**Table 3:** False negative and upgrading rates in targeted biopsy and standard prostate biopsy ISUP: International Society of Urological Pathology

		TARGETED BIOPSY (TB)					
		Benign	ISUP G1	ISUP G2	ISUP G3	ISUP G4	ISUP G5
STANDARD PROSTATE BIOPSY (SPB)	Benign	224	31	7	4	7	-
	ISUP G1	37	24	2	1	1	1
	ISUP G2	15	2	3	1	1	1
	ISUP G3	5	1	1	3	-	1
	ISUP G4	11	-	2	1	2	1
	ISUP G5	8	-	-	-	-	2

## Discussion

The results indicated that CB, which combined TB and SPB, had the highest csPCa detection rates in biopsy-naïve patients with PSA<10 ng/ml.

In one of our previous studies, we evaluated the clinical outcomes of our results of 80 patients that underwent FPB and we found that the csPCa detection rates for TB, SPB, and CB were 25.0%, 36.3%, and 43.8%, respectively [10]. In the present study, which is a continuation of that study, we found that the csPCa detection rates for TB, SPB, and CB in 400 biopsy-naïve patients with PSA<10 ng/ml were 25.0%, 31.8%, and 44.0%, respectively. A 2019 study evaluated 141 biopsy-naïve patients and reported the csPCa detection rates for TB, SPB, and CB as 51.8%, 53.9%, and 59.6%, respectively. The authors also indicated that CB was the most successful biopsy technique [14]. In that study, almost 80% of the patients had a PIRADS 4 or 5 lesion, while almost half of the patients in our study had a PIRADS 3 lesion. Accordingly, the cancer detection rates reported by that study could be higher than those of our study. Another study evaluated 131 biopsy-naïve patients with a median PSA value of 6.5 ng/ml and reported the csPCa detection rates for TB, SPB, and CB as 43.5%, 35.9%, and 49.6%, respectively. That study also noted that CB was the most successful biopsy technique [15]. Another recent study evaluated 317 biopsy-naïve patients and reported the csPCa detection rates for CB and TB as 57% and 50%, respectively [16]. In that study, unlike in our study, approximately 85% of the patients had a PIRADS 4 or 5 lesion. A systematic review examined 15 studies and reported the csPCa detection rates for TB and SPB as 23.6% and 33.3%, respectively [17]. Mozer et al., on the other hand, found similar overall PCa detection rates for SPB and TB (56.6% and 53.9%, respectively), while they found a significantly higher csPCa detection rate for TB compared to SPB (43.4% vs. 36.9%) [18]. Although these findings vary across studies, they suggest that CB is the most successful biopsy technique in biopsy-naïve patients.

One of the most important advantages of FPB is its high csPCa detection rates [6, 15, 19]. Valerio et al. reported that FPB had a significantly higher csPCa detection rate compared to SPB (33.3% vs. 23.6%, p<0.05) [6]. A 2020 study also noted that SPB (25%) had a higher isPCa rate compared to CB (19%) and TB (17%) [15]. In our study, in line with the literature, CB also had the highest csPCa detection rate.

Expectedly, our findings also indicated that cancer detection rates increased as the PIRADS score increased. CB detected cancer in almost 65% of PIRADS 5 lesions as opposed to 29% in PIRADS 3 lesions. Interestingly, TB was the least successful biopsy technique in PIRADS 3 lesions, even worse than SPB, whereas it was as successful as SPB in PIRADS 4 and 5 lesions. These findings could be attributed to the fact that as PIRADS score increases, lesions become larger and thus become more pronounced on fusion USG [20]. Nevertheless, CB was found to be the most successful biopsy technique in all PIRADS scores. Fujii et al. obtained similar findings to those of our study [15]. Of note, the authors indicated that SPB was more successful than TB in PIRADS 3 lesions while no significant difference was found between PIRADS 4 lesions and other lesions. Another study reported that the csPCa detection rates of TB in PIRADS 3, 4, and 5 lesions were 13%, 33%, and 89%, respectively [21]. Although the rates for PIRADS 3 and 4 lesions were consistent with those of our study, the csPCa detection rate reported for PIRADS 5 lesions was remarkably higher than that of our study.

In our previous study, we evaluated histopathological outcomes of patients that underwent radical prostatectomy following FPB and SPB and we found that FPB had lower upgrading rates compared to SPB [22]. In the present study, however, we compared SPB and TB by accepting CB as the golden standard and we found that TB had greater ISUP upgrading rates compared to SPB although no significant difference was established (19.6% vs. 13.7%, p=0.144). There are some studies in the literature presenting similar findings to those of our study [23, 24]. Siddiqui et al. evaluated FPB outcomes of 584 patients and reported that 38 additional patients were diagnosed with csPCa thanks to TB as opposed to seven additional patients that were diagnosed thanks to SPB [24]. On the other hand, studies have presented contradictory results regarding false negativity [15, 24]. In our study, the ratio of false negativity was higher for TB compared to SPB, which was inconsistent with the findings of Siddiqui et al. Nevertheless, our findings seem similar to those of Fujii’s results. Based on our findings regarding upgrading and false negative rates, we suggest that CB can be preferred in patients suspected with PCa.

Our study was limited in several ways. First, the study had a small patient population. Second, the number of patients with PIRADS 3 lesions was remarkably higher than that of patients with PIRADS 4 and 5 lesions, which could be attributed to the lack of our experience during the MRI examination of the first cases. Third, the number and ratio of patients with ISUP 1 was remarkably high, which could be ascribed to the fact that most of our patients had a gray-zone PSA level.

## Conclusions

Our findings indicated that FPB is the most successful biopsy technique in biopsy-naïve patients. Moreover, its higher cancer detection rates and its lower upgrading and false-negative rates implicate that SPB and TB should definitely be used in the form of CB. According to these findings, standard plus target biopsy (CB) after mpMRI seems to be the most ideal biopsy method, especially in patients with no previous biopsy history and PSA <10 ng/ml.

## References

[1] Bray F, Ferlay J, Soerjomataram I, Siegel RL, Torre LA, Jemal A (2018). Global cancer statistics 2018: GLOBOCAN estimates of incidence and mortality worldwide for 36 cancers in 185 countries. CA Cancer J Clin.

[2] Siddiqui MM, Rais-Bahrami S, Turkbey B (2015). Comparison of MR/ultrasound fusion-guided biopsy with ultrasound-guided biopsy for the diagnosis of prostate cancer. JAMA.

[3] Ahdoot M, Wilbur AR, Reese SE (2020). MRI-targeted, systematic, and combined biopsy for prostate cancer diagnosis. N Engl J Med.

[4] Vourganti S, Rastinehad A, Yerram N (2012). Multiparametric magnetic resonance imaging and ultrasound fusion biopsy detect prostate cancer in patients with prior negative transrectal ultrasound biopsies. J Urol.

[5] Mendhiratta N, Meng X, Rosenkrantz AB (2015). Prebiopsy MRI and MRI-ultrasound fusion-targeted prostate biopsy in men with previous negative biopsies: impact on repeat biopsy strategies. Urology.

[6] Valerio M, Donaldson I, Emberton M (2015). Detection of clinically significant prostate cancer using magnetic resonance imaging-ultrasound fusion targeted biopsy: a systematic review. Eur Urol.

[7] Elkhoury FF, Felker ER, Kwan L, Sisk AE, Delfin M, Natarajan S, Marks LS (2019). Comparison of targeted vs systematic prostate biopsy in men who are biopsy naive: the prospective assessment of image registration in the diagnosis of prostate cancer (PAIREDCAP) study. JAMA Surg.

[8] Yarlagadda VK, Lai WS, Gordetsky JB, Porter KK, Nix JW, Thomas JV, Rais-Bahrami S (2018). MRI/US fusion-guided prostate biopsy allows for equivalent cancer detection with significantly fewer needle cores in biopsy-naive men. Diagn Interv Radiol.

[9] Cattarino S, Forte V, Salciccia S (2019). MRI ultrasound fusion biopsy in prostate cancer detection: are randomized clinical trials reproducible in everyday clinical practice?. Urologia.

[10] Sönmez G, Tombul ŞT, İmamoğlu H, Akgün H, Demirtaş A, Tatlışen A (2019). Multiparametric MRI fusion-guided prostate biopsy in biopsy naive patients: preliminary results from 80 patients. Turk J Urol.

[11] Barentsz JO, Weinreb JC, Verma S (2016). Synopsis of the PI-RADS v2 guidelines for multiparametric prostate magnetic resonance imaging and recommendations for use. Eur Urol.

[12] Epstein JI, Egevad L, Amin MB, Delahunt B, Srigley JR, Humphrey PA (2016). The 2014 International Society of Urological Pathology (ISUP) consensus conference on Gleason grading of prostatic carcinoma: definition of grading patterns and proposal for a new grading system. Am J Surg Pathol.

[13] Arumainayagam N, Ahmed HU, Moore CM (2013). Multiparametric MR imaging for detection of clinically significant prostate cancer: a validation cohort study with transperineal template prostate mapping as the reference standard. Radiology.

[14] Preisser F, Theissen L, Wenzel M (2021). Performance of combined magnetic resonance imaging/ultrasound fusion-guided and systematic biopsy of the prostate in biopsy-naïve patients and patients with prior biopsies. Eur Urol Focus.

[15] Fujii S, Hayashi T, Honda Y (2020). Magnetic resonance imaging/transrectal ultrasonography fusion targeted prostate biopsy finds more significant prostate cancer in biopsy-naïve Japanese men compared with the standard biopsy. Int J Urol.

[16] van der Leest M, Cornel E, Israël B (2019). Head-to-head comparison of transrectal ultrasound-guided prostate biopsy versus multiparametric prostate resonance imaging with subsequent magnetic resonance-guided biopsy in biopsy-naïve men with elevated prostate-specific antigen: a large prospective multicenter clinical study. Eur Urol.

[17] Elwenspoek MM, Sheppard AL, McInnes MD (2019). Comparison of multiparametric magnetic resonance imaging and targeted biopsy with systematic biopsy alone for the diagnosis of prostate cancer: a systematic review and meta-analysis. JAMA Netw Open.

[18] Mozer P, Rouprêt M, Le Cossec C (2015). First round of targeted biopsies using magnetic resonance imaging/ultrasonography fusion compared with conventional transrectal ultrasonography-guided biopsies for the diagnosis of localised prostate cancer. BJU Int.

[19] Nassiri N, Beeder L, Nazemi A (2019). Step-by-step: fusion-guided prostate biopsy in the diagnosis and surveillance of prostate cancer. Int Braz J Urol.

[20] Weinreb JC, Barentsz JO, Choyke PL (2016). PI-RADS prostate imaging - Reporting and Data System: 2015, Version 2. Eur Urol.

[21] Shoji S, Hiraiwa S, Endo J (2015). Manually controlled targeted prostate biopsy with real-time fusion imaging of multiparametric magnetic resonance imaging and transrectal ultrasound: an early experience. Int J Urol.

[22] Demirtaş A, Sönmez G, Tombul ŞT, Demirtaş T, Akgün H (2019). Comparison of the upgrading rates of International Society of Urological Pathology grades and tumor laterality in patients undergoing standard 12-core prostate biopsy versus fusion prostate biopsy for prostate cancer. Urol Int.

[23] Calio BP, Sidana A, Sugano D (2018). Risk of upgrading from prostate biopsy to radical prostatectomy pathology-does saturation biopsy of index lesion during multiparametric magnetic resonance imaging-transrectal ultrasound fusion biopsy help?. J Urol.

[24] Siddiqui MM, Rais-Bahrami S, Truong H (2013). Magnetic resonance imaging/ultrasound-fusion biopsy significantly upgrades prostate cancer versus systematic 12-core transrectal ultrasound biopsy. Eur Urol.

